# Broadband Flexible Quantum Dots/Graphene Photodetectors

**DOI:** 10.3390/mi17010121

**Published:** 2026-01-16

**Authors:** Judy Z. Wu, Andrew Shultz

**Affiliations:** Department of Physics and Astronomy, University of Kansas, Lawrence, KS 66045, USA

**Keywords:** graphene, quantum dots, broadband photodetection, flexible, high sensitivity

## Abstract

Nanohybrids consisting of quantum dots and graphene (QD/graphene) provides a unique scheme to design quantum sensors. The quantum confinement in QDs enables spectral tunability, while that in graphene provides superior photocarrier mobility. The combination of them allows for broadband light absorption and high photoconduction gain that in turn leads to high photoresponsivity in QD/Gr nanohybrid photodetectors. Since the first QD/graphene photodetector was reported in 2012, intensive research has been conducted on this topic. In this paper, a review of the recent progress made on QD/Gr nanohybrid photodetectors will be provided. Among many applications, there will be a particular focus on broadband and flexible photodetectors, which make use of the inherent advantages of the QD/Gr nanohybrids. The remaining challenges and future perspectives will be discussed in this emerging topic area.

## 1. Introduction

The discovery of graphene by Geim and Novoselov in 2004 triggered explosive research on so-called two-dimensional atomic materials (2D materials) over the past two decades. Besides the numerous 2D materials discovered, van der Waals (vdW) heterostructures formed by stacking either different 2D materials or their nanohybrids with other low-dimensional materials such as quantum dots, nanowires, nanotubes, and nanostructures of a variety of shapes, sizes, and functionalities have emerged as a new platform in designing new materials with properties unprecedented by the conventional ones in bulk and film formats. The heterostructure nanohybrids differ from conventional materials in several aspects. One such aspect is the strong quantum confinement in the constituents due to their low dimensionality, which leads to new electronic structures and physical properties that are not available in conventional materials. For example, graphene—a monolayer of carbon atoms arranged in a flat 2D honeycomb lattice [1]—exhibits a Dirac-cone electronic structure with a linear dispersion, resulting in massless fermions (both electrons and holes) with an extraordinary mobility of up to 200,000 cm^2^·V^−1^·s^−1^ at room temperature [1,2]. On the other hand, interfaces represent another unique aspect of vdW heterostructures, which are often used as a unique design parameter to obtain new physical properties and functionality. Bilayer graphene stacked with selected rotational angles is an excellent example, in which the two sheets of graphene can exhibit “Moire” patterns as carbon atoms on the two sheets form periodic alignment to enable new properties of twist-electronics, including superconductivity. Therefore, vdW heterostructure nanohybrids provide a fascinating platform in the exploration of new materials and device structures that are not readily available in conventional cases. These nanohybrids devices have a variety of applications in electronics and optoelectronics [3,4].

**Figure 1:**
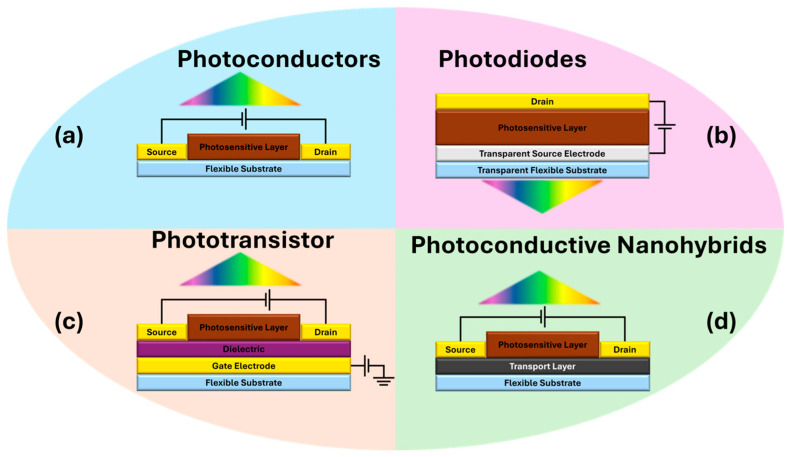
Device structures of four common types of flexible photodetectors: (**a**) photoconductors, (**b**) photodiodes, (**c**) phototransistors and (**d**) photoconductive nanohybrids.

Photodetectors are important optoelectronic devices with wide applications such as security systems, communications, smartphone cameras, environmental monitoring, night vision, medical imaging, etc. [5]. Figure 1 exhibits a few commonly used examples of conventional photodetector device structures including two-terminal photoconductors (with a photosensitive semiconductor layer) and photodiodes (two semiconductor layers: photosensitive and window) and three terminal phototransistors (a gate electrode is adopted). The nanohybrid photodetectors implement a charge transfer channel to the photosensitive semiconductor layer and may operate as two-terminal (similar to photoconductor) or three-terminal (similar to phototransistor). The channel typically has higher carrier mobility than that of the photosensitive layer to allow for reduced charge recombination and hence enhanced external quantum efficiency (EQE). Higher EQE is critical to achieve high photoresponsivity (*R*) in photodetectors [6]. Therefore, nanohybrids that implement semiconductor quantum dots and a high-mobility graphene channel (QD/graphene) provide a unique platform to explore photodetectors with high responsivity in the broadband spectrum, enabled by the spectral tunability of quantum dots. Furthermore, QD/graphene nanohybrids are inherently flexible for applications requiring flexible, wearable, curved photodetectors and imagers [6,7,8,9,10].

## 2. Quantum Physics in QD/Graphene Nanohybrids PDs

QD/graphene nanohybrids are quantum devices. The strong quantum confinements in both QDs and graphene make QD/graphene nanohybrids and their interface introduces new quantum physics for the design of quantum devices with functionalities and performance without precedence in conventional counterparts, as detailed in the following. VdW heterojunction nanohybrids of QD/graphene combine the advantages of the spectral tunability in QDs [11] and high charge carrier mobility in graphene to enable the creation of quantum sensors for different applications. One of the most important applications of QD/graphene nanohybrids is photodetectors. In these nanohybrids, QDs absorb incident lights to allow for broadband photodetection by selecting QDs of different bandgaps in ultraviolet, visible, and infrared spectra.

**Figure 2:**
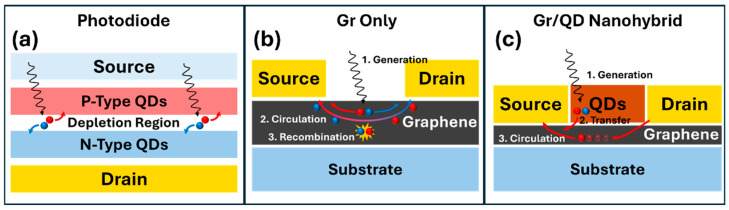
The detection mechanisms of QD-based (**a**) photodiodes, (**b**) Gr-only photoconductors, and (**c**) Gr/QD nanohybrids. Red/blue dots and arrows represent positively/negatively charged carriers and the direction of movement, respectively.

Figure 2 compares the optoelectronic processes in conventional semiconductor photodiodes, including those consisting of p- and n-types of QDs (Figure 2a), graphene-only (Figure 2b), and QD/graphene (Figure 2c) photodetectors. In conventional photodetectors, light absorption, photocarrier generation, and transfer all occur in the photosensitive semiconductor layer (Figure 1a–c and Figure 2a). Therefore, photocarrier transfer is limited by carrier mobility (µ, typically in the range of few to 100 s of cm^2^/V-s in crystalline semiconductor films [12] while several orders of magnitude lower in QD layers due to additional surface defects and QD-QD junctions) in the photosensitive semiconductor layer. If graphene is used to replace the photosensitive layer (Figure 2b), the tradeoff for its higher µ is the low light absorption in monolayer graphene, despite the high absorption of ~2.3%/sheet in the near-UV to mid-THz spectral range [13]. While various light absorption-enhancing mechanisms such as plasmonic nanostructures have been explored, low *R* up to a few mA/W seems uncompetitive with conventional photodetectors. This issue can be addressed in QD/graphene nanohybrids, which adopt a QD layer as the photosensitive layer and a graphene channel of high carrier mobility for charge transport, which enables high photoconductive gain and hence high *R* (details in next section). Furthermore, the adoption of the graphene channel leads to a decoupled photocurrent from the dark current associated with thermal excitation. This means that the photoconductive gain would enhance only the photocurrent, not both the photocurrent and the dark current as in the conventional photodetectors. In addition, the QD/graphene nanohybrids are inherently flexible. The progress made in the growth of wafer-size graphene and in transferring graphene to other substrates, including flexible ones, provides scalability of devices based on QD/graphene nanohybrids [1,14,15,16,17,18,19,20,21]. This means that the established microfabrication processes can be applied for the fabrication of graphene-based devices and circuits [10,21,22,23,24,25,26,27,28,29,30].

### 2.1. Photoconductive Gain

In 2D monolayer graphene of ~0.34 nm in thickness, electrons and holes are subjected to strong quantum confinement within the 2D atomic sheet. This results in a Dirac-cone electronic band structure [1] with linear dispersion, which means that the carriers are massless fermions with high Fermi velocity *v*_F_~10^6^ m/s, 1/300 of the speed of light in a vacuum. This leads to high carrier mobility (µ) up to 10^6^ cm^2^·V^−1^·s^−1^ for both electrons and holes [1,2,15,31,32]. The high carrier mobility implies that graphene would serve as an excellent channel material with reduced carrier recombination. On the other hand, the quantum confinement in QDs can lead to spectral tunability as the bandgap (*E*_g_) is inversely proportional to the QD diameter, leading to a blue shift in spectral range with decreasing QD dimension. Furthermore, enhanced light–solid interaction or excitonic absorption is anticipated from the excitonic electronic structure in QDs due to the quantum confinement.

In QD/graphene nanohybrids, QDs are responsible for light absorption, while graphene is used as the channel for photo-generated carriers. Specifically, the exciton generated in QDs upon light absorption will be dissociated into holes and electrons, with one kind of charge carriers transferred to graphene with the assistance of the built-in electric field at the QD/graphene interface due to the band-edge alignment. Therefore, QD/graphene nanohybrids combine the advantages of their constituents, including excitonic light absorption with spectral tunability and high carrier mobility in the electric transfer channel. Since the electrical conductivity of the graphene channel can be tuned by the gate electric field [33], the other kind of charges remain in the QD, which provides so-called photo-gating on graphene, and the channel conductance change is detected for extraction of the photo response in QD/graphene nanohybrid photodetectors.

PbS QD/graphene nanohybrids were the first QD/graphene photodetectors reported by Konstantatos et al. in 2012 [9]. Many works on QD/graphene photodetectors with a high photoconductive gain (*G*) exceeding 10^10^ and hence high responsivity (*R*) have been reported [9,34,35,36,37,38,39,40,41]. It should be noted that the high gain is proportional to the ratio of exciton lifetime in QDs and carrier transit time in the graphene channel. This predicts that the gain and hence photoresponsivity (*R* ∝ *G*) depends on the graphene channel length (*L*) as *R* ∝ *L*^−2^. This theoretical prediction has been confirmed experimentally by Shultz et al. recently on an array of PbS QD/graphene nanohybrid photodetectors with variable *L* in the range 10–150 µm in a broad optical spectrum ranging from ultraviolet (UV), to visible (Vis), to infrared (IR) [42]. This experimental confirmation illustrates that QD/graphene heterojunction nanohybrids are indeed governed by the quantum physics associated with the constituents (Figure 2c).

Various figures of merit are used to characterize the performance of a photodetector, including quantum efficiency, responsivity, noise, signal-to-noise ratio, specific detectivity, etc. [43]. Responsivity (R) is used to quantify the signal output (photocurrent, *I_ph_*) from the photodetector per unit optical power (*P_in_*) and is expressed as

(1) $$\begin{array}{c} R=\frac{I_{ph}}{P_{in}}. \end{array}$$

The photocurrent originates from the photo-generated carriers. In a photodiode, photocurrent is determined by the following formula:

(2) $$\begin{array}{c} I_{ph}=G\eta q\frac{P_{in}}{\hbar \omega } \end{array}$$

where η is the quantum efficiency or the ratio between the number of electron–hole pairs generated per incident photon, ℏ is the Planck constant, and ω is the light frequency. G is photoconductive gain that is proportional to the ratio between the exciton life time (*τ_c_*) and the carrier transit time (*τ_t_*) between the source and drain electrodes. It should be noted that *τ_c_* in QDs is considerably larger than that in semiconductor bulks or films due to quantum confinement in the QDs, which means that reduced G values are expected if QDs are replaced with films or continuous layers of semiconductors [44,45]. On the other hand, *τ_t_* defined as $\;\tau_{t}=\frac{L^{2}}{V_{sd}\;\mu }$, where *L* is the QD/graphene pixel channel length, *µ* is the carrier mobility, and *V_sd_* is the applied bias voltage between source and drain electrodes could be very short due to the high carrier mobility of graphene *µ*. Consequently, the QD/graphene nanohybrids are expected to have a large G exceeding 10^10^. It should be noted that the high gain is at the cost of the slower response speed, typically on the order of *τ_c_* [46]. The speed could be further reduced when charge traps associated with various defects at the QD-QD and QD/graphene interfaces [47].

### 2.2. Geometry and Noise Considerations

The previous discussion presented the dependence of the *R* ∝ *L*^−2^ relationship derived from the quantum physics in QD/graphene nanohybrids. Experimental verification requires controlling nanohybrids’ pixel quality and uniformity to allow for extraction of the geometry effect, which has been not been performed until recently [42]. It should be noted that this geometry effect provides a convenient avenue to achieve high *R* values that, on the other hand, may be misleading to the realistic sensitivity of a QD/graphene photodetector. Therefore, a comprehensive study of *R*, noise, and specific detectivity (D*) is necessary. *R* is simply a ratio of the generated photocurrent to the incident light power and thus does not account for the noise present in the detector, which may pose a significant hurdle to signal distinguishability, depending on the detector design. In the QD/graphene architecture, the graphene is the conductive channel, possessing many orders of magnitude more conductance than a QD film, which leads to the graphene introducing nearly all of the noise present in a QD/graphene device, with negligible contributions from the QD film. The noise dominance of graphene has been experimentally confirmed, with three main contributors to the noise in graphene: thermal, shot, and 1/f noises.

**Figure 3:**
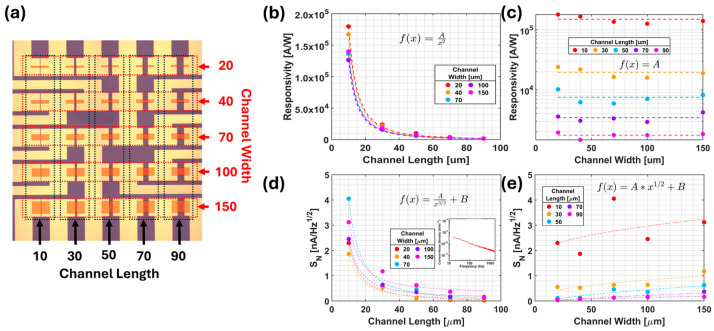
(**a**) An array of QD/graphene channel with varied channel geometry. (**b**,**c**) Responsivity vs. channel length and width, respectively. (**d**,**e**) Noise current density vs. channel length and width, respectively. The inset of (**d**) shows a typical noise spectral density of graphene. Adapted with permission from Ref. [42]. Copyright 2024 American Chemical Society.

The noise present in a photodetector needs to be accounted for in order to present an accurate picture of the sensitivity of a photodetector. D* is the accepted figure of merit for photodetectors, defined as

(3) $$\begin{array}{c} D^{*}=\frac{R\sqrt{LW∆f}}{I_{N}},\; \end{array}$$

where *W* is the channel width, Δ*f* is the bandwidth, and *I_N_* is the noise current. D* provides an excellent measure of detector sensitivity, with a built-in quantification of the signal-to-noise ratio (*R/I_N_*), as well as geometric considerations associated with shot noise ($\sqrt{LW}$). Often, when direct measurements of the noise are not possible, an estimate of noise (assuming shot noise dominant) is made as $I_{sh}=\sqrt{2qIG∆f}$, where *I* is the average DC current. This estimate does not account for the significant contribution by 1/f noise (see the inset of Figure 3d for a typical noise spectrum), highlighting the need for empirical quantification of noise for accurate detector performance reporting. It should be noted that this formula includes the internal gain of the photodetector, the exclusion of which would lead to a much-overestimated D*, which has been experimentally confirmed recently by Shultz et al., showing that $I_{N}\sim W^{1/2}L^{-3/2}$ on an array of 5 × 5 PbS QD/graphene photodetectors with variable *L* (10–90 µm), *W* (20–150 µm), and electrode contact areas as shown Figure 3a. The *R* ∝ *L*^−2^ relationship is confirmed (Figure 3b), while negligible *W*-dependence is observed (Figure 3c). Meanwhile, $I_{ph}\sim L^{-2}$, the noise current is $\sim L^{-3/2}$(Figure 3d), and $\sim W^{1/2}$ (Figure 3e). This provides a means to achieve enhanced signal-to-noise by simply optimizing channel geometry, such as by reducing the channel length.

### 2.3. Balance Light Absorption and Carrier Transfer

Nanostructures such as QDs are well known for their large surface-to-volume ratio, and the surface is where absorption and chemical reactions occur. This means that defects and adsorbents on the surfaces and interfaces of nanohybrids can sensitively impact the performance of the nanohybrids in general [48,49]. In QD/graphene nanohybrids, there exist multiple surfaces and interfaces, including QD and graphene surfaces, the interface between them, and the interface between them and substrates and the environment. These surfaces and interfaces play a critical role in governing light absorption, exciton dissociation, and charge carrier transport, which determine the device performance of QD/graphene nanohybrids. For example, in QD/graphene nanohybrids, when photons are absorbed by QDs and excitons (electron–hole pairs) are generated, the band-edge alignment across the QD/graphene interface leads to a built-in electric field to facilitate the dissociation of excitons to free electrons and holes. In addition, this built-in field can assist the transfer of one kind of charge carriers from QDs to graphene, leading to so-called photo-gating on the graphene channel as the photoresponse.

When the QD/graphene interface is contaminated by even a monolayer of unwanted molecules attached to graphene, such as polar molecules from air (H_2_O and O_2_), chemical residues (from device fabrication), and defect states formed on QDs and/or insulating ligands attached to the surface of QDs, this optoelectronic process would be negatively affected, which could lead to poor performance or even failure of the QD/graphene nanohybrid photodetectors. For example, in HgTe QD/graphene photodetectors for middle-wave IR (MWIR) detection, Gong et al. discovered that HgTe QDs have surface defects sensitively affected by the QD synthesis processes [41]. No MWIR photoresponse is detectable if Tellurium atoms are highly deficient on the surface of the HgTe QDs, since the defective QD surface states serve as charge traps preventing charge transfer from QDs to graphene. By addressing this issue using organic solvent in QD synthesis, a high *R* and hence a high specific detectivity (D*), a figure of merit, up to 2.4 × 10^11^ Jones was obtained in the MWIR spectrum at room temperature, owing to the high *G* > 10^7^.

**Figure 4:**
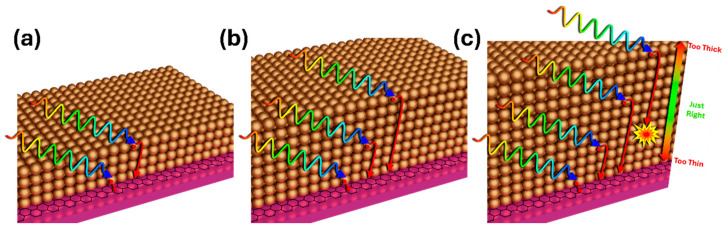
Light absorption and carrier transfer in a QD/graphene nanohybrid with varied layer thicknesses: (**a**) Thin QD layer with lower light absorption. (**b**) Optimal QD layer thickness to allow for maximum incident light absorption and all photocarriers collected. (**c**) QD layer too thick, with a substantial fraction of photocarrier recombined before completing transfer to graphene.

In many cases, QDs are synthesized in organic solvents. One of the advantages is the presence of ligands. The attached ligands on the QD surface not only assist in the suspension of the formed QDs but also provide effective passivation to control the chemical reaction at the QD surface. Such control could protect the QDs from decomposition in ambient conditions by minimizing the exposure and hence the decomposition of QDs. Most of the ligands, however, are insulating, which means that charge transfer across QD-QD and QD/graphene interfaces may be blocked by a layer of insulating ligands in QD/graphene nanohybrids that rely on photo-generated carriers to transfer through the QD layer and across the QD/graphene interface. Therefore, ligand exchange has been developed to replace the more insulating ligands from the synthesis solvents with more conducting ligands to establish an electric conducting network in QD/graphene nanohybrids. In QD/graphene nanohybrids, the QD layer may be formed through multiple QD coatings for optimal light absorption. On the other hand, the total thickness of the QD layer may not go beyond the threshold to allow photo-generated carriers to reach the graphene channel. As exhibited schematically in Figure 4, multiple QD coatings are often necessary in order to reach a compromise between light absorption and carrier transport, which implies that multiple ligand exchanges may be necessary. In a study reported by Shultz et al., a ligand exchange following each QD coating was found to provide the best photoresponse [50]. In contrast, if the ligand exchange was carried out after QD multilayer deposition, a photoresponse comparable to one QD layer was observed, which illustrates the critical importance of controlling QD-QD and QD/graphene interfaces for efficient photocarrier transfer to graphene.

## 3. Recent Progress in QD/Graphene Nanohybrids PDs

### 3.1. Device Designs for Broadband Flexible Photodetection

**Figure 5:**
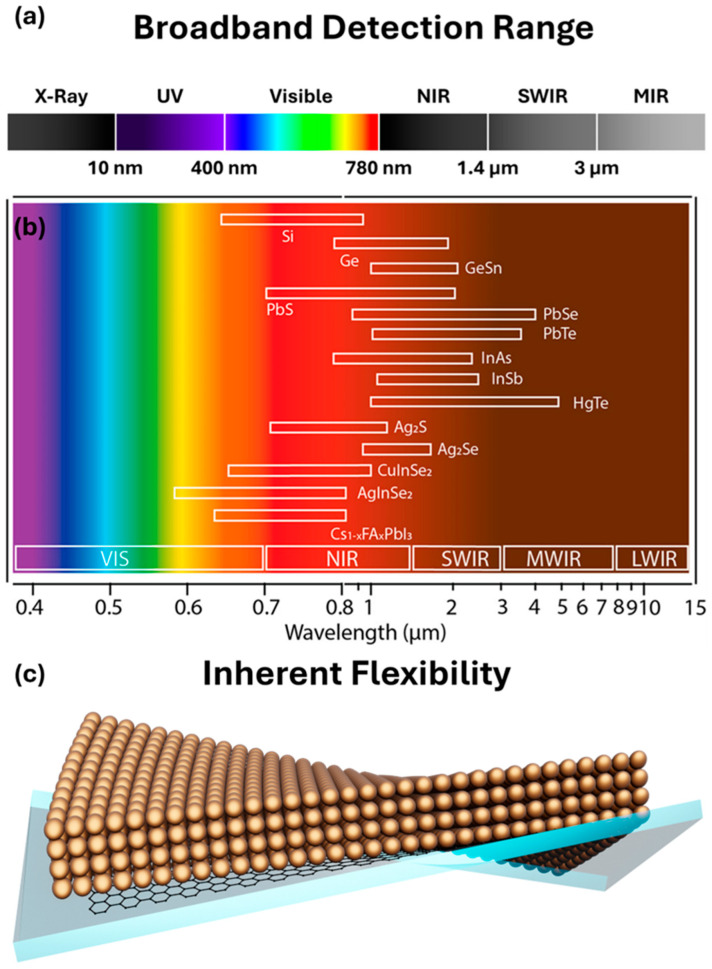
(**a**) Detection spectrum of QD/Gr nanohybrids. (**b**) Emission spectral range of various QDs of varied size. (**c**) Flexibility granted by the thin nature of graphene and fluid-like deformability of QDs. Adapted with permission from Ref. [11]. Copyright 2019 American Chemical Society.

The spectral range of QD/graphene nanohybrids is determined by the bandgap (*E_QD_*) of QDs. The strong quantum confinement in semiconductor QDs leads to advantages not only in the high photoconductive gain in the QD/graphene nanohybrids discussed in Section 2.1, but also in the tunability of the spectral range through control of their shape, dimension, functionality (core/shell QDs), and carrier doping [51]. The *E_QD_* dependence on the QD’s size and shape can be expressed in the Brus equation [52,53,54]:

(4) $$E_{QD}=E_{bulk}+\frac{{2h}^{2}}{d^{2}}\left(\frac{1}{m_{e}}+\frac{1}{m_{h}}\right)-\frac{3.6e^{2}}{4\pi \varepsilon \varepsilon_{0}d}$$

where *E_bulk_* is the bandgap of the corresponding bulk semiconductor, *h* is Planck’s constant, *d* is the diameter of the QDs, *m_e_* and *m_h_* are the effective masses of electron and hole, respectively, *ε* is the dielectric constant, $\varepsilon_{0}$ is the vacuum permittivity, and *e* is the electron charge. The second and third term of Equation (4) represent the quantum confinement effect and Coulomb interaction, respectively. Figure 5 exhibits the spectral range and QD candidates for broadband flexible photodetectors. Therefore, the absorption spectral range can be tuned by the size of QDs, which is directly related to the tunable bandgap of the QDs [55]. In addition, the bandgap of the QDs can also be tuned in a broad range by varying their chemical composition. For example, the absorption spectral range of all-inorganic metal-halide perovskites CsPbX_3_ NCs can cover the entire visible range by changing the elements X (Cl, Br, I) [56].

Localized surface plasmonic resonance (LSPR) can also be induced in QDs via carrier doping, or in other plasmonic nanostructures placed in proximity to the QD/graphene nanohybrids. LSPR could provide a light-trapping effect to enhance the light absorption of QDs and shift the spectral range via exciton–plasmon coupling [57,58,59]. In the former, the light wavelength selectivity could be determined by the LSPR frequency of the plasmonic sensitizers, providing a viable wavelength tuning mechanism [51]. Besides doping semiconductor nanocrystals to produce LSPR for broadband and enhanced absorption, metal/semiconductor core/shell structure design is another efficient way to improve the light absorption at a limited active shell thickness with low absorption capacity. A template modulated colloidal approach for synthesizing metal core (AuCu) and metal-halide perovskite shell (CsPbCl_3_) nanocrystals was reported [60]. Enhanced light absorption was observed in the CsPbCl_3_ shell with thickness of 2–4 nm, ascribed to the LSPR AuCu core with an average diameter of 7.1 nm. The LSPR AuCu core-induced light absorption of the perovskite shell enabled a remarkable 30-times-enhanced photoresponse in core/shell perovskite QD/graphene nanohybrid photodetectors, compared to the counterparts with perovskite-only QDs. Plasmonic metal nanostructures may be placed in proximity to the nanohybrid photodetectors for the benefit of light trapping, which can be quantified using Finite-Difference Time-Domain (FDTD) simulation, and enhanced light absorption in QDs [61]. A recent work by Alamri et al. combined the plasmonic effects from TMD nanodiscs grown on graphene and Ag nanoparticles (AgNPs) using an in vacuo process, producing a seven-fold-enhanced photoresponse [62].

The graphene used in QD/graphene nanohybrids is typically monolayer for its distinctive electronic structure. As the number of layers increases, graphene evolves into regular graphite. An interesting candidate in the graphene–graphite family is double-layer graphene, which may have so-called AB stacking, with specially arranged atom alignment between the two graphene monolayers, other alignments, or even no alignment, by stacking two monolayer graphene in an uncontrolled manner. The advantage of double-layer graphene is the reduced effect from adsorbed species on the graphene surface, which typically lead to p-doping (by air molecules for example), or increased carrier scattering, and hence reduced carrier mobility. In order to evaluate the effect, a comparative study of QD/graphene nanohybrids with either monlayer or double-layer graphene as the channel materials was reported by Olson et al., and no significant difference was observed in broadband photodetection in the 400–1500 nm wavelength range [63]. However, removing adsorbed species on graphene was found to be important in reducing the noise of graphene, which dominates the noise of QD/graphene nanohybrids, and enhancing charge transfer across the QD/graphene interface, which in turn enhances *R*. For example, ozone treatment of graphene for a few minutes was found to be effective to remove adsorbed species on graphene [64].

### 3.2. Performance of Broadband Photodetection (R, D*, Speed)

Using a diverse set of QDs, many QD/graphene nanohybrid photodetectors have been explored in the last decade or so using various low-cost, scalable methods including spin-coating [9], inkjet printing [30,37,38,40,65,66,67,68,69,70,71], and direct growth [10,65,68,72]. Besides individual devices, imaging arrays have also been obtained, including the demonstration of QD/graphene focal plane arrays with Si-based readout circuits [35]. Table 1 summarizes a few examples of rigid and flexible nanohybrid photodetectors A general trend is that the performance of the former is considerably higher, which means that further improvement in flexible device fabrication is important in future research. Note that a few conventional photodetectors are also included in Table 1 for performance comparison with QD/graphene nanohybrid photodetectors. For example, MWIR photodetectors consisting of HgTe QDs only (conventional) and HgTe QD/graphene nanohybrids are both included in Table 1. A distinctive difference is the higher D* in the latter without cooling. At room temperature, Gong et al. reported D* up to 1.0 × 10^12^ Jones (2.25 µm) to 5.1 × 10^10^ Jones (4 µm) [41]. This is in contrast to 10^10^ Jones (4 µm) at 220 K and 4.0 × 10^11^ Jones (4.5 µm) at 85 K [73] using HgTe QD films. In fact, the high MWIR D* in uncooled HgTe QD/graphene nanohybrids represents the first demonstration that comparable D* in cooled MWIR conventional photodetectors can be achieved without cooling, a unique merit of decoupled dark currents and photocurrents in QD/graphene nanohybrids [41].

## 4. Applications of QD/Gr PDs

### 4.1. Broadband Imaging

**Figure 6:**
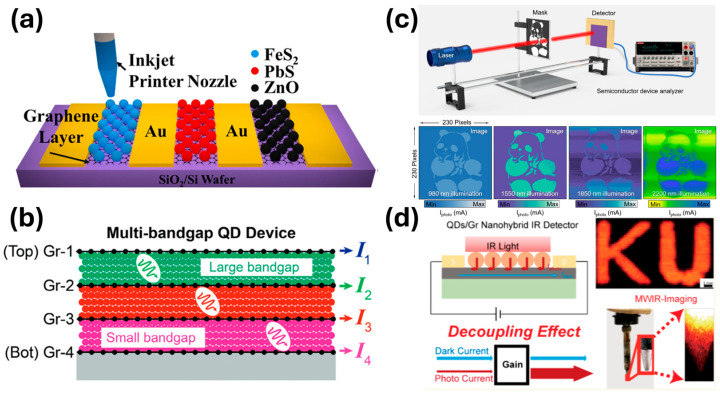
(**a**) Pixelated multi-QD photodetector for color detection [65]. (**b**) Tandem structured multi-QD photodetector for color detection [91]. (**c**) Imaging with QD/graphene photodetectors in the NIR to SWIR range [92]. (**d**) Imaging with QD/graphene photodetectors in the visible–SWIR–MIR range [90]. Adapted with permission from Ref. [65]. Copyright 2019 American Chemical Society. Adapted with permission from Ref. [91]. Copyright 2024 John Wiley & Sons. Adapted with permission from Ref. [92]. Copyright 2024 American Chemical Society. Copyright 2025 American Chemical Society.

Table 1 and Figure 6 include a few examples of broadband photodetectors reported recently using different schemes of one kind or multiple kinds of semiconductor active layers for light absorption. For example, Gong et al. reported a broadband photosensitizer based on FeS_2_ nanocubes that demonstrated strong LSPR effect, illustrated by the enhanced absorption and widened spectral range covering the UV–visible–SWIR (SWIR: short-wave IR) broadband [40]. This LSPR FeS_2_/graphene nanohybrid photodetector exhibits an efficient broadband photoresponse, with responsivities reaching 1.08 × 10^6^ A/W in the whole UV–visible–SWIR range at a low operational voltage of 0.1 V. In the latter case, both pixelated (Figure 6a) and tandem (Figure 6b) photodetectors have been explored to demonstrate broadband and multi-color photodetection and imaging.

### 4.2. Flexible PDs/Array for Imaging

Over the past decade, flexible photodetectors have generated significant interest for their potential use in wearable and deformable electronics. Materials choices become nontrivial, with the need for consistent performance while subjected to various deformations. Nanomaterials, such as single to few 2D layers, 1D nanotubes, and 0D quantum dots are well-suited to this task due to their inherent flexibility at small dimensions. Devices fabricated on various substrates have been reported, including polymer [93,94,95,96,97], paper [98,99,100], human hair [101], and mica [102,103]. Various photosensitive nanomaterials have been utilized in photodetection including layered 2D sheet(s) [104,105,106,107], nanotubes [108,109,110,111,112], quantum dots [85,113,114,115], and hybrids [65,86,91,92,100]. Device fabrication must consider the thermal budgets and chemical resilience of the materials in use to ensure the suitability of the processes used. Popular fabrication techniques include dry/wet transfer, spin coating, dip-coating, ink-jet printing, e-beam evaporation, sputtering, pulsed laser deposition, physical/chemical vapor deposition, and atomic layer deposition. Exciting progress has been made towards nanomaterial-based flexible photodetectors for imaging with comparable or better performance to conventional rigid imagers.

**Figure 7:**
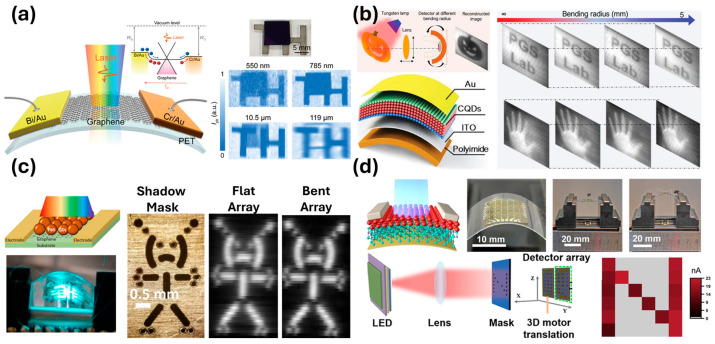
Four examples of flexible photodetectors from recent works. (**a**) A graphene-only flexible imager. (**b**) A QD-only flexible imager. (**c**) A QD/graphene nanohybrid imager. (**d**) A QD/MXene nanohybrid imager. Adapted with permission from Ref. [116]. Copyright 2024 John Wiley & Sons. Adapted with permission from Ref. [117]. Copyright 2019 John Wiley & Sons. Adapted with permission from Ref. [86]. Copyright 2024 American Chemical Society. Adapted with permission from ref. [118]. Copyright 2023 American Chemical Society.

An imaging device consisting only of asymmetric electrodes and graphene was reported recently by Liu et al. (Figure 7a), fabricated on a PET substrate for flexible imaging [116]. The zero bandgap of graphene and asymmetric electrodes provided a unique photothermoelectric avenue for visible–NIR–SWIR–MIR–LWIR photodetection. A QD-only approach consisting of a 400 nm thick HgTe QD layer as the sensitizer has been reported by Tang et al. on a polyimide substrate (Figure 7b), achieving a D* of 3.3 × 10^10^ Jones at a wavelength of 1000 nm, as well as flexible imaging capability [117]. Shultz et al. recently reported a QD–graphene nanohybrid fabricated on a PET substrate demonstrating a D* of ~10^10^ Jones in the UV–Vis spectrum when under tensile stress (Figure 7c) [86]. This device exhibited imaging capability while under different bending conditions. Zhu et al. recently demonstrated a flexible nanohybrid consisting of PbS QDs with an MXene carrier transport layer achieving a D* of 10^11^ at a wavelength of 980 nm (Figure 7d) [118].

### 4.3. Curved PDs and Imagers

**Figure 8:**
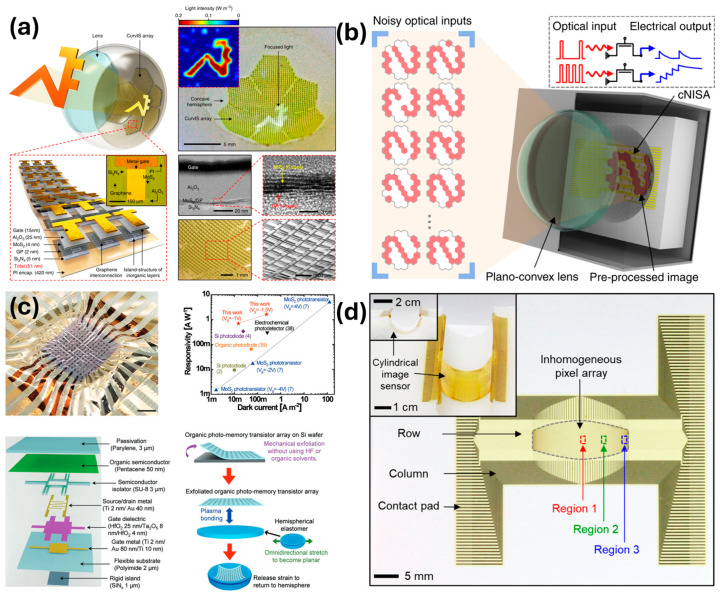
Four examples of curved photodetector arrays from recent works. (**a**) An array of MoS_2_-Graphene curved phototransistors. (**b**) An array of MoS_2_-pV3D3 curved phototransistors. (**c**) An array of organic semiconductor based curved phototransistors. (**d**) An inhomogeneous array of Si photodiodes for undistorted imaging. Adapted with permission under a Creative Commons CC License from Ref. [119]. Adapted with permission under a Creative Commons CC License from Ref. [120]. Adapted with permission from Ref. [121]. Copyright 2023 John Wiley and Sons. Adapted with permission under a Creative Commons CC License from Ref. [122].

Curved photodetectors have gained increased interest recently due to the widespread availability of nanomaterials and simple fabrication techniques for devices employing these materials. One benefit of curved photodetectors is their ability to conform to the spherical image location formed by spherical lenses, thus reducing the need for extra lenses in the optical system to correct for this effect. A wide viewing angle and 360° imaging are additional benefits afforded by curved imagers. The main difficulty encountered in the widespread replacement of rigid imagers is fabricating high-density and large-pixel-number arrays due to the lack of on-chip conductance pathways that Si-based rigid imaging arrays rely upon. To address this issue, recent works have focused on flexible devices incorporating large numbers of nanomaterial-based photodetectors. Choi et al. recently demonstrated a 12 × 12 pixel MoS_2_-graphene heterostructured spherical imaging array with flexible printed circuit board readout for a bio-inspired eye prosthesis (Figure 8a) [119]. Similar work with a 31-pixel MoS_2_-pV3D3 heterostructured array of photodetectors in a spherical shape exhibited a neuromorphic imager for potential machine vision applications (Figure 8b) [120].

Establishing electrical connections is often a source of difficulty in producing large numbers of detectors due to the inability to design built-in circuitry into the substrate itself. To solve this, Kim et al. recently demonstrated a 12 × 12 hemispherical array based on an organic semiconductor with liquid metal connections for array readout (Figure 8c) [121]. The transition from a flat plane to a curved surface can lead to deformations in the image due to uneven stretching/compression of pixels based on location and curvature. Recent work by Choi et al. demonstrated a nonuniform distribution of pixels to counterbalance the distortion normally present in a cylindrically curved array of 4096 pixels (Figure 8d) [122].

## 5. Summary and Future Perspectives

In summary, since the first report of PbS QD/graphene photodetectors in 2012 [9], significant progress has been made in the exploration of QD/graphene nanohybrid photodetectors and imagers for broadband photodetection on rigid and flexible substrates. The performance on individual devices using a large variety of QDs of different spectral ranges is impressive. This progress has triggered interest in further research on and development of QD/graphene nanohybrid photodetectors in several aspects. One aspect is to further expand the spectral range, which is defined by the cutoff or bandgap of semiconductor QDs used in the nanohybrids. Innovations are certainly important and are required to break the conventional bandgap-determined spectral range. Furthermore, the development of arrays of QD/graphene photodetectors has emerged as a focus for imaging applications, taking advantage in particular of their inherent flexibility for flexible and curved imagers. In addition, multi-color QD/graphene nanohybrid photodetectors demand further research, despite the progress made in tandem-structured and multi-pixelated devices. Each has pros and cons, but this must be taken into consideration when scaling up to large imaging arrays.

Regarding the prospective applications of QD/graphene nanohybrids, several major challenges must be addressed. A major challenge is in atomic-scale control of the QD/graphene interface, which directly affects exciton dissociation and charge transfer. Since different QDs may have different surface states, the development of a universal surface or interface engineering protocol is difficult. For example, an atomically thin Zn acetate layer on ZnO QDs can completely block the charge transfer from ZnO QDs to graphene and lead to a negligible photoresponse to UV light. When this surface layer is removed, a photoresponse improved by orders of magnitude can be obtained. On the other hand, many QDs, such as PbS, HgTe, and CsPbX_3_ (X = Cl, Br, I), have surface states due to the presence of defects and dangling bonds on the QDs surface, which lead to not only QD decomposition in ambient conditions but also charge traps at the QD/graphene interface, degrading both photoresponse and response speed. Ligand exchange has been adopted for the passivation of such surface states and hence the improvement of QD-QD and QD/graphene interfaces, as illustrated by the improved photodetector performance, especially at longer wavelengths. It should be noted that the surface and interface passivation schemes may not work universally on different nano/graphene nanohybrids. This means that a continuous effort is necessary to develop new interface engineering approaches in the exploration of new QD/graphene nanohybrids. Another challenge is in scaling up for large arrays and integration with the readout circuits that can be used for flexible and curved QD/graphene nanohybrid imagers. Achieving high device yield and performance uniformity is certainly an important task towards commercialization of nano/graphene heterojunction nanohybrids. Nevertheless, the exploration of new QD/graphene nanohybrid photodetectors and imagers for flexible broadband photodetection will continue to be the research focus worldwide for new quantum devices and applications.

## Figures and Tables

**Table 1:** Performance of nanohybrid photodetectors on rigid and flexible substrates. QD—quantum dots, NC—nanocrystals, Gr—graphene, HgTe—made from Hg acetate precursor, HgTe*—made from HgCl_2_ precursor. The *R* and D* values were collected at 300 K unless otherwise indicated.

Rigid Device	Spectral Range	*R* (A/W)	D* (Jones)	Response Time (s)	Reference
ZnO QD/Gr	UV	9.9 × 10^8^	7.5 × 10^14^ (at 340 nm)	5/85	Ref. [37]
ZnSe/ZnS core/shell QD/Gr	UV	2 × 10^3^	-	0.52	Ref. [74]
WS_2_ QD/Gr	UV	3.8 × 10^3^	1.6 × 10^13^ (at 365 nm)	2.04	Ref. [75]
CsPbCl_3_ QD/Gr	UV-vis	3.5 × 10^6^	10^13^ (at 390 nm)	0.3	Ref. [39]
CsPbBr_3−x_I_x_ NC/Gr	Vis	8.2 × 10^8^	2.4 × 10^16^ (at 405 nm)	3.65	Ref. [76]
AuCu/CsPbCl_3_ core/shell QD/Gr	UV-Vis	20–6.5 (300–600 nm)			Ref. [60]
FeS_2_/PbS QD/Gr	UV-NIR	3.27 × 10^6^	4.89 × 10^11^ (at 500 nm)	15	Ref. [38]
PbS QD/Gr	Vis-SWIR	10^7^	7 × 10^13^ (at 532 nm)/10^12^ (at 1.55 µm)	10 × 10^−2^	Refs. [9,35]
Cu_3−x_P QD/Gr	Vis-SWIR	9.34	5.98 × 10^12^ (at 405 nm)	1	Ref. [77]
HgTe* QD	MWIR	1.62-NA	4 × 10^11^(4.5 µm, 85 K)–10^10^ (4.0 µm, 220 K)		Ref. [73]
HgTe* NC	SWIR	1.2 × 10^3^	2.0 × 10^12^ (1.55 µm, 200 K)	2.2 × 10^−5^	Ref. [78]
HgTe* QD/Gr	SWIR	900	6 × 10^8^ (at 2.5 µm, 80 K)		Ref. [79]
HgTe QD/Gr	SWIR-MWIR	5.3 × 10^4^–2.6 × 10^3^	1.0 × 10^12^ (2.25 µm)—5.1 × 10^10^ (4 µm)	10^−3^–10^−3^	Ref. [41]
Si (B-doped) QD/Gr	UV-MWIR	10	10^5^ (at 3 µm)	-	Ref. [80]
HgTe* NC/HgSe QD	MWIR	6	2.0 × 10^8^ (200 K)	<10^4^	Ref. [81]
HgTe* QD/MoS_2_	SWIR	5 × 10^3^	10^12^ (2 µm)	4 × 10^−3^	Ref. [82]
Ti_2_O_3_ QD/Gr	MWIR-LWIR	>120	7 × 10^8^ (at 10 µm)	10^−3^	Ref. [83]
**Flexible Devices**	**Spectral range**	***R*** **(A/W)**	**Flexibility (radius, cycles)**	**Imaging**	**Reference**
CsPbCl_3_ NC	UV	8.1	NA, 1600		Ref. [84]
CsPbBr_3_ QD	UV-vis	10.1	NA, 1000		Ref [85]
CsPbCl_3_ QD/Gr	UV-vis	3.5 × 10^6^	NA, 25		Ref. [39]
PbS QD/Gr array	Vis-NIR	5–90	>5 mm	Direct/scanning	Ref. [86]
P3HT:PCBM/Gr	Vis-NIR	5.8 × 105	5 mm, 100		Ref. [87]
CdSe/ZnS core/Shell on polymer array	Vis-multicolor	1.5 × 10^−5^	Stretchable and flexible	3-color imaging	Ref. [88]
PbS QD array	X-ray-NIR	0.4 (600 nm) and 2 × 10^5^ μC Gy−1 cm^−3^ (X-ray)	flexible	Direct imaging	Ref. [89]
HgTe QD/Gr	UV-MWIR	0.65–10^−3^	>5 mm	scanning	Ref. [90]

## Data Availability

Not applicable.
